# Rates of change of pons and middle cerebellar peduncle diameters are diagnostic of multiple system atrophy of the cerebellar type

**DOI:** 10.1093/braincomms/fcae019

**Published:** 2024-02-21

**Authors:** Christopher D Stephen, Mark Vangel, Anoopum S Gupta, Jason P MacMore, Jeremy D Schmahmann

**Affiliations:** Ataxia Center, Massachusetts General Hospital and Harvard Medical School, Boston, MA 02114, USA; Cognitive Behavioral Neurology Unit, Massachusetts General Hospital and Harvard Medical School, Boston, MA 02114, USA; Laboratory for Neuroanatomy and Cerebellar Neurobiology, Department of Neurology, Massachusetts General Hospital and Harvard Medical School, Boston, MA 02114, USA; Biostatistics Center, Massachusetts General Hospital and Harvard Medical School, Boston, MA 02114, USA; Martinos Center for Biomedical Imaging, Department of Radiology, Massachusetts General Hospital and Harvard Medical School, Boston, MA 02129, USA; Ataxia Center, Massachusetts General Hospital and Harvard Medical School, Boston, MA 02114, USA; Cognitive Behavioral Neurology Unit, Massachusetts General Hospital and Harvard Medical School, Boston, MA 02114, USA; Ataxia Center, Massachusetts General Hospital and Harvard Medical School, Boston, MA 02114, USA; Cognitive Behavioral Neurology Unit, Massachusetts General Hospital and Harvard Medical School, Boston, MA 02114, USA; Laboratory for Neuroanatomy and Cerebellar Neurobiology, Department of Neurology, Massachusetts General Hospital and Harvard Medical School, Boston, MA 02114, USA; Ataxia Center, Massachusetts General Hospital and Harvard Medical School, Boston, MA 02114, USA; Cognitive Behavioral Neurology Unit, Massachusetts General Hospital and Harvard Medical School, Boston, MA 02114, USA; Laboratory for Neuroanatomy and Cerebellar Neurobiology, Department of Neurology, Massachusetts General Hospital and Harvard Medical School, Boston, MA 02114, USA

**Keywords:** ataxia, biomarker, deep phenotyping, neuroimaging

## Abstract

Definitive diagnosis of multiple system atrophy of the cerebellar type (MSA-C) is challenging. We hypothesized that rates of change of pons and middle cerebellar peduncle diameters on MRI would be unique to MSA-C and serve as diagnostic biomarkers. We defined the normative data for anterior–posterior pons and transverse middle cerebellar peduncle diameters on brain MRI in healthy controls, performed diameter–volume correlations and measured intra- and inter-rater reliability. We studied an Exploratory cohort (2002–2014) of 88 MSA-C and 78 other cerebellar ataxia patients, and a Validation cohort (2015–2021) of 49 MSA-C, 13 multiple system atrophy of the parkinsonian type (MSA-P), 99 other cerebellar ataxia patients and 314 non-ataxia patients. We measured anterior–posterior pons and middle cerebellar peduncle diameters on baseline and subsequent MRIs, and correlated results with Brief Ataxia Rating Scale scores. We assessed midbrain:pons and middle cerebellar peduncle:pons ratios over time. The normative anterior–posterior pons diameter was 23.6 ± 1.6 mm, and middle cerebellar peduncle diameter 16.4 ± 1.4 mm. Pons diameter correlated with volume, *r* = 0.94, *P* < 0.0001. The anterior–posterior pons and middle cerebellar peduncle measures were smaller at first scan in MSA-C compared to all other ataxias; anterior–posterior pons diameter: Exploratory, 19.3 ± 2.6 mm versus 20.7 ± 2.6 mm, Validation, 19.9 ± 2.1 mm versus 21.1 ± 2.1 mm; middle cerebellar peduncle transverse diameter, Exploratory, 12.0 ± 2.6 mm versus 14.3 ±2.1 mm, Validation, 13.6 ± 2.1 mm versus 15.1 ± 1.8 mm, all *P* < 0.001. The anterior–posterior pons and middle cerebellar peduncle rates of change were faster in MSA-C than in all other ataxias; anterior–posterior pons diameter rates of change: Exploratory, −0.87 ± 0.04 mm/year versus −0.09 ± 0.02 mm/year, Validation, −0.89 ± 0.48 mm/year versus −0.10 ± 0.21 mm/year; middle cerebellar peduncle transverse diameter rates of change: Exploratory, −0.84 ± 0.05 mm/year versus −0.08 ± 0.02 mm/year, Validation, −0.94 ± 0.64 mm/year versus −0.11 ± 0.27 mm/year, all values *P* < 0.0001. Anterior–posterior pons and middle cerebellar peduncle diameters were indistinguishable between Possible, Probable and Definite MSA-C. The rate of anterior–posterior pons atrophy was linear, correlating with ataxia severity. Using a lower threshold anterior–posterior pons diameter decrease of −0.4 mm/year to balance sensitivity and specificity, area under the curve analysis discriminating MSA-C from other ataxias was 0.94, yielding sensitivity 0.92 and specificity 0.87. For the middle cerebellar peduncle, with threshold decline −0.5 mm/year, area under the curve was 0.90 yielding sensitivity 0.85 and specificity 0.79. The midbrain:pons ratio increased progressively in MSA-C, whereas the middle cerebellar peduncle:pons ratio was almost unchanged. Anterior–posterior pons and middle cerebellar peduncle diameters were smaller in MSA-C than in MSA-P, *P* < 0.001. We conclude from this 20-year longitudinal clinical and imaging study that anterior–posterior pons and middle cerebellar peduncle diameters are phenotypic imaging biomarkers of MSA-C. In the correct clinical context, an anterior–posterior pons and transverse middle cerebellar peduncle diameter decline of ∼0.8 mm/year is sufficient for and diagnostic of MSA-C.

## Teaser

In a 20-year longitudinal clinical and imaging study, Massachusetts General Hospital Ataxia Center neurologists discover a way to diagnose multiple system atrophy of the cerebellar type (MSA-C) with certainty using brain MRI. A decline of ∼0.8 mm/year in the diameter of the pons or middle cerebellar peduncles is diagnostic of MSA-C.

## Graphical Abstract

**Graphical Abstract:**
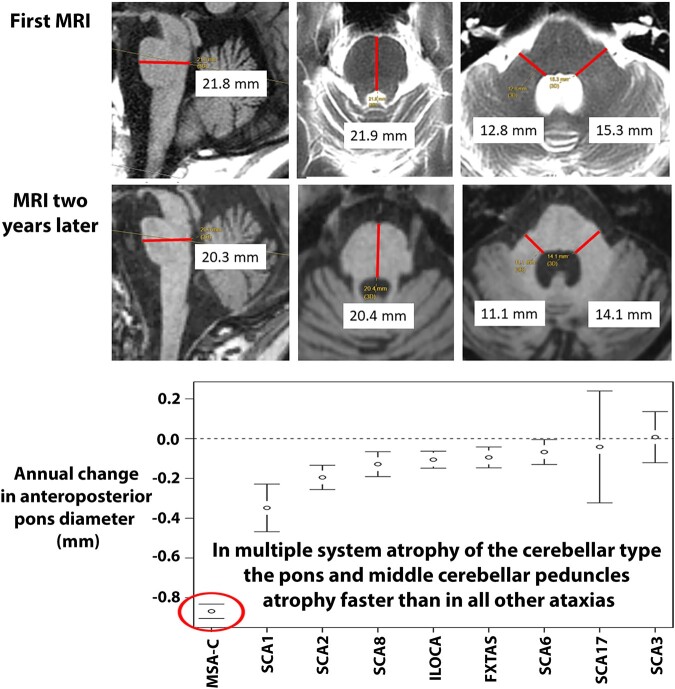

## Introduction

Multiple system atrophy (MSA) is a sporadic, adult-onset neurodegenerative synucleinopathy characterized by the combination of autonomic neuropathy and either parkinsonism (MSA-P^1^) or cerebellar ataxia (MSA-C), although phenotypic overlap is not uncommon.^2,3^ The disease manifestations evolve inexorably, causing progressive disability and death usually within a decade of motor symptom onset.^2,4-7^

The diagnosis of Definite MSA is confirmed at autopsy, characterized by α-synuclein-positive oligodendroglial cytoplasmic inclusions and neuronal loss in the basal ganglia, brainstem and cerebellum.^4,8^ Diagnosis during life is challenging because there is no single confirmatory test.

The differentiation of MSA-C from other causes of late-onset sporadic or non-familial ataxia^5,6^ can be vexing, and MSA-P is difficult to distinguish from Parkinson’s disease (PD) and other forms of atypical parkinsonism.^9^ In the 2008 consensus criteria,^4^ Possible versus Probable MSA are differentiated by clinical severity of autonomic dysfunction only, while suggestive features of Possible MSA (not required for Probable MSA) include the imaging finding of pontocerebellar atrophy, putaminal rim hyperintensity and hot cross bun sign (HCBS) on anatomic MRI, and increased diffusivity of the putamen and middle cerebellar peduncle (MCP) on diffusion-weighted imaging.^10^ However, none of these findings is specific to MSA.^11-14^ Concerns over clinical heterogeneity and insensitivity to detection of early disease^3^ prompted the Movement Disorders Society to propose new criteria that include imaging features (not further defined) but this applies only to the highest probability diagnosis of Clinically Established MSA.^15^

In our previous study of 65 patients with Possible, Probable and subsequently proven Definite MSA-C,^6^ we concluded that a sporadic onset, insidiously developing cerebellar syndrome in midlife, with autonomic features of otherwise unexplained bladder dysfunction with or without erectile dysfunction in males, and atrophy of the cerebellum, brainstem and MCP pointed strongly to a diagnosis of MSA-C. Other clinical features of REM sleep behaviour disorder (RBD) and postural hypotension confirmed the diagnosis, while extrapyramidal findings, corticospinal tract signs and pathologic laughing and crying were helpful but not necessary for diagnosis. Inherent in our conclusions, therefore, was the statement that imaging findings of cerebellar and brainstem volume loss accompany the clinical presentation.

Here, we test our hypothesis derived from clinical observation over the past 20 years, which imaging biomarkers are diagnostic of MSA-C. Cerebellar atrophy in MSA-C is rapid and dramatic^16,17^ resulting principally from loss of deep and folial white matter,^17^ but it is not practicable to measure these neuroimaging changes in the routine clinical setting. We therefore focused on pons and MCP dimensions, as these are readily identifiable and measurable at the point-of-care by healthcare providers, using conventional clinical MRI. We predicted that the rate of change of the diameters of these structures, as a proxy for volumetric analysis, could be used clinically to diagnose MSA-C with certainty during life.

## Methods

This study was approved by the Mass General Brigham Institutional Review Board (IRB).

### Study cohorts

#### Healthy controls

We acquired normative imaging data from 73 healthy individuals in the 1000 Functional Connectomes, as described below.

#### Exploratory cohort

We performed a retrospective review of patients seen in the Massachusetts General Hospital (MGH) Ataxia Center between January 2002 and December 2014. Two cohorts were studied.

##### MSA cohort

We identified 88 patients with MSA-C: 74 met consensus criteria^4^ for Possible/Probable MSA, and 14 patients who died during the Exploratory phase met autopsy criteria for Definite MSA-C. Nine of the 74 Possible/Probable MSA-C patients died after the completion of the Exploratory phase of the study, underwent autopsy, and were confirmed as Definite MSA-C.

##### Cerebellar ataxia cohort—not MSA

We studied 44 patients with spinocerebellar ataxia (SCA) types 1, 2, 3, 5, 6, 7, 8 and 17; 13 with Friedreich’s ataxia (FA), 6 with fragile X-associated tremor/ataxia syndrome (FXTAS); and 15 who we designated as idiopathic late-onset cerebellar ataxia (ILOCA) after extensive negative evaluation including polyglutamine and other repeat disorders and exome sequencing (see Table 1). None of these ataxia control patients had isolated downbeat nystagmus, recently identified as an indicator of SCA27B.^18^

**Table 1:** Exploratory cohort: demographic details and AP Pons and MCP diameters at first scan

Diagnosis	Male:female (% male)	Age of motor onset (years) Mean ± SD	Age at first scan (years) Mean ± SD	Patients with multiple scans (*N*, %)	Mean AP Pons (axial) mm	Mean AP Pons (sagittal) mm	Mean MCP mm
MSA-C Definite/Possible/Probable (*n* = 88)	53:35 (60.2)	56.4 ± 8.2	60.0 ± 8.1	60 (68.2)	19.3 ± 2.6	19.9 ± 2.5	12.0 ± 2.6
MSA-C Definite (*n* = 14)	8:6 (57.1)	55.8 ± 8.7	60.2 ± 7.4	7 (50.0)	18.0 ± 2.2	18.9 ± 2.8	11.1 ± 2.5
MSA-Possible/Probable (*n* = 74)	45:29 (60.8)	56.6 ± 8.2	59.9 ± 8.3	53 (71.6)	19.5 ± 2.7	20.0 ± 2.5	12.1 ± 2.6
Other ataxias (*n* = 78)	49:29 (62.8)	37.8 ± 17.9	48.2 ± 15.8	22 (28.2)	20.7 ± 2.6	21.6 ± 2.4	14.3 ± 2.1
FA (*n* = 13)	7:6 (53.9)	15.5 ± 10.7	36.6 ± 16.9	0	20.2 ± 2.0	22.1 ± 1.7	14.5 ± 1.5
ILOCA (*n* = 15)	10:5 (66.7)	38.3 ± 11.8	48.4 ± 11.8	7 (46.7)	22.1 ± 2.3	21.9 ± 2.2	15.1 ± 2.2
SCA1 (*n* = 8)	4:4 (50.0)	44.3 ± 11.0	52.3 ± 10.3	1 (12.5)	18.6 ± 2.2	19.4 ± 1.8	12.3 ± 1.9
SCA2 (*n* = 5)	3:2 (60.0)	26.4 ± 14.7	33.9 ± 9.7	2 (40.0)	17.7 ± 4.2	18.1 ± 3.9	11.8 ± 3.3
SCA3 (*n* = 12)	7:5 (58.3)	42.0 ± 14.0	48.6 ± 12.0	1 (8.3)	20.1 ± 2.0	21.1 ± 1.7	14.0 ± 1.5
SCA5 (*n* = 1)	0:1 (0)	48.0	50.7	0 (0)	22.1	22.7	16.0
SCA6 (*n* = 8)	4:4 (50.0)	48.4 ± 10.8	58.8 ± 7.8	2 (25.0)	23.2 ± 1.4	23.3 ± 1.4	16.0 ± 1.1
SCA7 (*n* = 3)	3:0 (100)	32.0 ± 28.0	37.7 ± 31.2	0 (0)	18.6 ± 0.7	20.7 ± 1.5	13.8 ± 0.9
SCA8 (*n* = 5)	3:2 (60.0)	43.6 ± 15.5	56.8 ± 15.6	2 (40.0)	23.0 ± 1.8	23.0 ± 1.9	15.5 ± 1.7
SCA17 (*n* = 2)	2:0 (100)	25.5 ± 14.8	35.3 ± 21.0	1 (50.0)	21.5 ± 0.2	21.6 ± 1.3	15.6 ± 0.4
FXTAS (*n* = 6)	6:0 (100)	64.0 ± 9.9	66.2 ± 10.2	6 (100)	20.4 ± 1.8	20.7 ± 1.8	13.3 ± 1.6

MSA-C, multiple system atrophy of the cerebellar type; FA, Friedreich’s ataxia; ILOCA, idiopathic late-onset cerebellar ataxia; SCA, spinocerebellar ataxia; FXTAS, fragile X-associated tremor/ataxia syndrome.

#### Validation cohort

We prospectively studied three cohorts of patients in the MGH Ataxia Center between January 2015 and December 2021. Demographic data collected included age, sex, diagnosis, age of motor symptom onset and age at which each scan was performed.

##### MSA cohort

There were 49 patients with MSA-C (30 Probable, 19 Possible). Whereas 13 patients died during this period (12 Probable, 1 Possible), none came to autopsy and therefore we could not make the consensus criteria designation of Definite MSA. We also included 13 patients with MSA-P (12 Probable and one who died, with Definite MSA-P confirmed at autopsy).

##### Cerebellar ataxia cohort—not MSA

We studied 99 patients with a range of acquired, genetic and sporadic cerebellar ataxias, as listed in Table 2.

**Table 2:** Validation cohort: demographic details and AP Pons and MCP diameters at first scan

Diagnosis	Male:female (% male)	Age of motor onset (years) Mean ± SD	Age at first scan (years) Mean ± SD	Patients with multiple scans (*N*, %)	Mean AP Pons (axial) mm	Mean MCP mm
MSA-C Possible/Probable (*n* = 49)	26:23 (53.1)	59.1 ± 8.6	60.9 ± 8.4	44 (89.8)	19.9 ± 2.1	13.6 ± 2.1
MSA-C Probable (*n* = 30)	18:12 (60.0)	60.1 ± 8.4	62.9 ± 7.8	26 (86.7)	19.7 ± 2.3	13.3 ± 2.3
MSA-C Possible (*n* = 19)	8:11 (42.1)	56.8 ± 7.9	57.7 ± 8.5	18 (94.7)	20.2 ± 1.6	14.0 ± 1.6
MSA-P (*n* = 13)	9:4 (69.2)	64.5 ± 7.1	66.4 ± 7.4	9 (69.2)	21.5 ± 1.2	15.9 ± 1.4
Ataxia (*n* = 99)^a^	49:50 (49.5)	41.1 ± 19.9	51.5 ± 15.4	49 (49.5)#	21.1 ± 2.1	15.1 ± 1.8
PD (*n* = 79)	47:32 (59.5)	64.4 ± 10.6	67.5 ± 10.7	24 (30.4)	22.9 ± 1.4	17.5 ± 1.1
Atypical parkinsonism (*n* = 40)	21:19 (52.5)	67.8 ± 11.5	72.2 ± 10.2	6 (15.0)	22.2 ± 2.0	16.8 ± 1.5
Essential tremor (*n* = 31)	16:15 (51.6)	59.4 ± 15.0	63.8 ± 12.7	1 (3.2)	22.5 ± 1.7	17.3 ± 0.8
Idiopathic and genetic dystonias (*n* = 30)	11:19 (36.7)	46.1 ± 21.7	52.7 ± 18.4	1 (8.3)	22.5 ± 1.8	16.9 ± 1.2
Movement disorder NOS (*n* = 9)	2:7 (22.2)	63.8 ± 21.7	66.4 ± 9.3	33 (33.3)	22.0 ± 1.1	16.6 ± 1.0
Other neurological disorder (*n* = 57)	26:31 (45.6)	55.9 ± 21.1	60.5 ± 19.9	14 (24.6)	22.2 ± 1.4	16.6 ± 1.1
FND (*n* = 79)	30:49 (38.0)	47.3 ± 15.3	51.5 ± 15.9	19 (24.1)	22.6 ± 1.4	17.1 ± 1.2
Drug-induced movement disorder (*n* = 11)	2:9 (18.2)	54.2 ± 15.3	56.3 ± 15.3	3 (27.3)	22.4 ± 2.4	16.5 ± 1.9
Non-neurological (*n* = 18)	6:12 (33.3)	51.3 ± 21.1	53.2 ± 18.0	2 (11.1)	22.3 ± 1.5	17.2 ± 1.4

MSA-C, multiple system atrophy of the cerebellar type; MSA-P, multiple system atrophy of the parkinsonian type; PD, Parkinson’s disease; NOS, not otherwise specified; FND, functional neurological disorder. ^a^Ataxia cohort included: sporadic ataxia *n* = 2; alcoholic cerebellar degeneration *n* = 5; Langerhan’s cell histiocytosis-related neurodegeneration with ataxia *n* = 1; autoimmune ataxia *n* = 4; acquired (tumours, stroke, hypoxia) *n* = 4; ataxia with oculomotor apraxia type 4 (AOA4) *n* = 1; cerebellar ataxia with neuropathy and vestibular areflexia syndrome (CANVAS) *n* = 1; episodic ataxia *n* = 2; Friedreich’s ataxia (FA) *n* = 5; late-onset FA *n* = 4; fragile X-associated tremor/ataxia syndrome (FXTAS) *n* = 4; Gerstmann–Sträussler–Scheinker disease (GSS) *n* = 1; late-onset Tay–Sachs *n* = 7; late-onset Sandhoff disease *n* = 3; PNPLA6-related disorder *n* = 1; spinocerebellar ataxia type 1 (SCA1) *n* = 1; SCA2 *n* = 5; SCA3 *n* = 12; SCA6 *n* = 4; SCA7 *n* = 1; SCA8 *n* = 3; SCA12 *n* = 1; SCA17 *n* = 1; SCA28 *n* = 1; SCA34 *n* = 1; SCA36 *n* = 1; SCA48 *n* = 1; 4H syndrome with ataxia *n* = 1; likely genetic ataxia NOS *n* = 21. # Ataxia cohort with repeat scans (*n* = 49) comprised: Acquired ataxia (tumors, stroke, hypoxia) *n* = 3, Alcoholic cerebellar degeneration *n* = 2, Ataxia with oculomotor apraxia type 4 (AOA4) *n* = 1, Likely genetic ataxia NOS *n* = 11, GSS *n* = 1, autoimmune ataxia *n* = 2, CANVAS *n* = 1, Episodic ataxia *n* = 1, FA *n* = 2, late-onset FA *n* = 2, FXTAS *n* = 3, Late-onset Tay-Sachs *n* = 2, LCH-related neurodegeneration with ataxia *n* = 1, PNPLA6-related disorder *n* = 1, SCA2 *n* = 3, SCA3 *n* = 2, SCA6 *n* = 4, SCA8 *n* = 1, SCA17 *n* = 1, SCA28 *n* = 1, SCA 34 *n* = 1, SCA36 *n* = 1, Sporadic ataxia *n* = 2.

##### Movement Disorders Cohort without cerebellar ataxia

To test the specificity of our putative imaging biomarker, we studied pontine measures in patients followed in a general movement disorders clinic who did not have cerebellar ataxia. This cohort comprised 79 patients with Parkinson’s disease (PD); 40 non-MSA atypical parkinsonism [progressive supranuclear palsy (PSP), corticobasal syndrome, dementia with Lewy Bodies]; 31 essential tremor; 30 idiopathic and genetic dystonia; 9 other primary movement disorders (degenerative chorea, downbeat nystagmus without ataxia, hereditary spastic paraplegia); 57 other neurological disorders (multiple sclerosis, normal pressure hydrocephalus, anterior horn cell disease, multifactorial gait disorder, myelopathy); 79 functional neurological disorder (FND); 11 drug-induced movement disorders and 18 non-neurological conditions (see Table 2).

#### Clinical measure of ataxia in the MSA cohorts

To determine the rate of clinical change over the course of each patient’s trajectory, and to compare this with the brainstem measures, we scored ataxia severity using the Brief Ataxia Rating Scale (BARS). The BARS^19^ is a five-item rating scale that scores the canonical manifestations of the cerebellar motor syndrome: gait, heel-to-shin, finger-to-nose, speech and oculomotor performance. The scale is scored from 0 to 30: higher scores indicate greater motor impairment. The BARS is tightly correlated with the Scale for Assessment and Rating of Ataxia^20^ and the International Cooperate Ataxia Rating Scale,^21^ and has been replicated and validated.^22,23^ A revised version with half-point measures describing deficits with greater granularity has been validated in clinical assessments^24,25^ and in quantitative observer-independent sensor studies.^26-31^ In late MSA-C, parkinsonism can become severe and mask ataxia. We use maximum BARS scores when tasks are no longer possible regardless of whether this was related to parkinsonism or ataxia.

### Brain imaging

#### Normative data in healthy controls

We acquired normative data for the brainstem measurements using the 1000 Functional Connectomes Project in the neuroimaging data repository of the Neuroimaging Tools and Resources Collaboratory, Project ID: fcon_1000 (www.nitrc.org). We studied 10 subjects each in the 2nd, 4th, 7th and 8th decades, 12 in the 3rd, 11 in the 5th, nine in the 6th, and one individual in the 9th decade (see Table 3). Images were viewed in MRIcron, a cross-platform NIfTI format image viewer in the nitrc platform. At the time these measures were taken in 2015, the platform did not yet have a function tool for line measurement. We developed a method to derive linear measures of the pons in the axial and sagittal planes and the diameter of the MCPs. We identified the points of interest on the pons and MCP in the sagittal and axial planes (sagittal plane, *y* + *z*; axial plane, *x* + *y*), determined the voxel space between them translated into mm space, and calculated the hypotenuse representing the diameters.

**Table 3:** Normative values for the diameters of the AP Pons measured in the sagittal and axial planes, and the transverse MCP diameters

Age (years)	*N*	Sagittal	SD	Axial	SD	MCP (L)	SD	MCP (R)	SD
10 to 19	10	23.2	1.6	22.6	1.9	16.3	1.2	15.9	1.6
20 to 29	12	23.4	1.4	23.3	1.5	15.7	1.3	15.3	1.3
30 to 39	10	23.7	1.1	23.7	1.5	17.1	1.8	17.3	2.0
40 to 49	11	24.3	1.8	24.4	1.8	16.4	1.5	16.2	1.3
50 to 59	9	24.5	1.2	24.7	1.7	17.0	0.9	17.6	1.2
60 to 69	10	23.7	1.2	23.7	1.4	17.2	1.8	16.7	1.3
70 to 79	10	23.9	1.7	23.2	1.2	16.0	1.4	16.1	1.1
80 to 89	1	22.5		22.1		15.6		14.9	
Total	**73**	**23.8**	**1.4**	**23.6**	**1.6**	**16.5**	**1.5**	**16.4**	**1.5**

	Pons AP	MCP
Normal	23.6	16.4
−1 SD	22.0	15.0
−2 SD	20.4	13.6
−3 SD	18.8	12.2
−4 SD	17.2	10.8
−5 SD	15.6	9.4
−6 SD	14.0	8.0
−7 SD	12.4	6.6
−8 SD	10.8	5.2

Normative values for the AP Pons and MCP diameters derived from the 1000 Functional Connectomes Project in the neuroimaging data repository of the Neuroimaging Tools and Resources Collaboratory, Project ID: fcon_1000 (www.nitrc.org), and a *Z*-score guide to the degree of atrophy for a single case using standard deviations below the mean. In the *Z*-score guide, the MCP value of 16.4 mm represents the average of the right and left MCP measures. SD, standard deviation; L, left; R, right; AP, anteroposterior; MCP, middle cerebellar peduncle. The mean values for the entire Normative cohort are highlighted in the bottom row in bold for emphasis.

#### Images analysed in the patient cohorts

Brain MRI studies were available on all patients in the MGH electronic health record (Amicas PACS viewer until April 2016; thereafter, eUnity PACS viewer v6.10.2.489, Client Outlook, Waterloo, Canada). We performed the measurements on standard MGH desktop computers available to practitioners in the clinic setting, using the ruler tool in viewing programs in Amicas and eUnity. We analysed MPRAGE or SPGR sequences when available because these 3D T_1_-weighted images have high spatial resolution and grey matter–white matter differentiation that makes them ideally suited for anatomical study.^32^ If these were not available, we used standard T_1_-weighted and T_2_-weighted sequences. We avoided fluid attenuated inversion recovery (FLAIR) sequences in which the brainstem boundaries are not as crisply defined.

In the Exploratory cohort, we analysed scans at every available time-point in all patients. Measures were performed with 0.1 mm accuracy.

In the Validation cohort, we analysed scans at every available time-point in the MSA-C/MSA-P cohort. For all other patients, the first and last scans were assessed from symptom onset through December 2021. These measures were performed with 0.5 mm accuracy, to reflect differences in the accuracy of measurement tools with different viewing programs.

### Quantitative outcome measures

**Figure 1:**
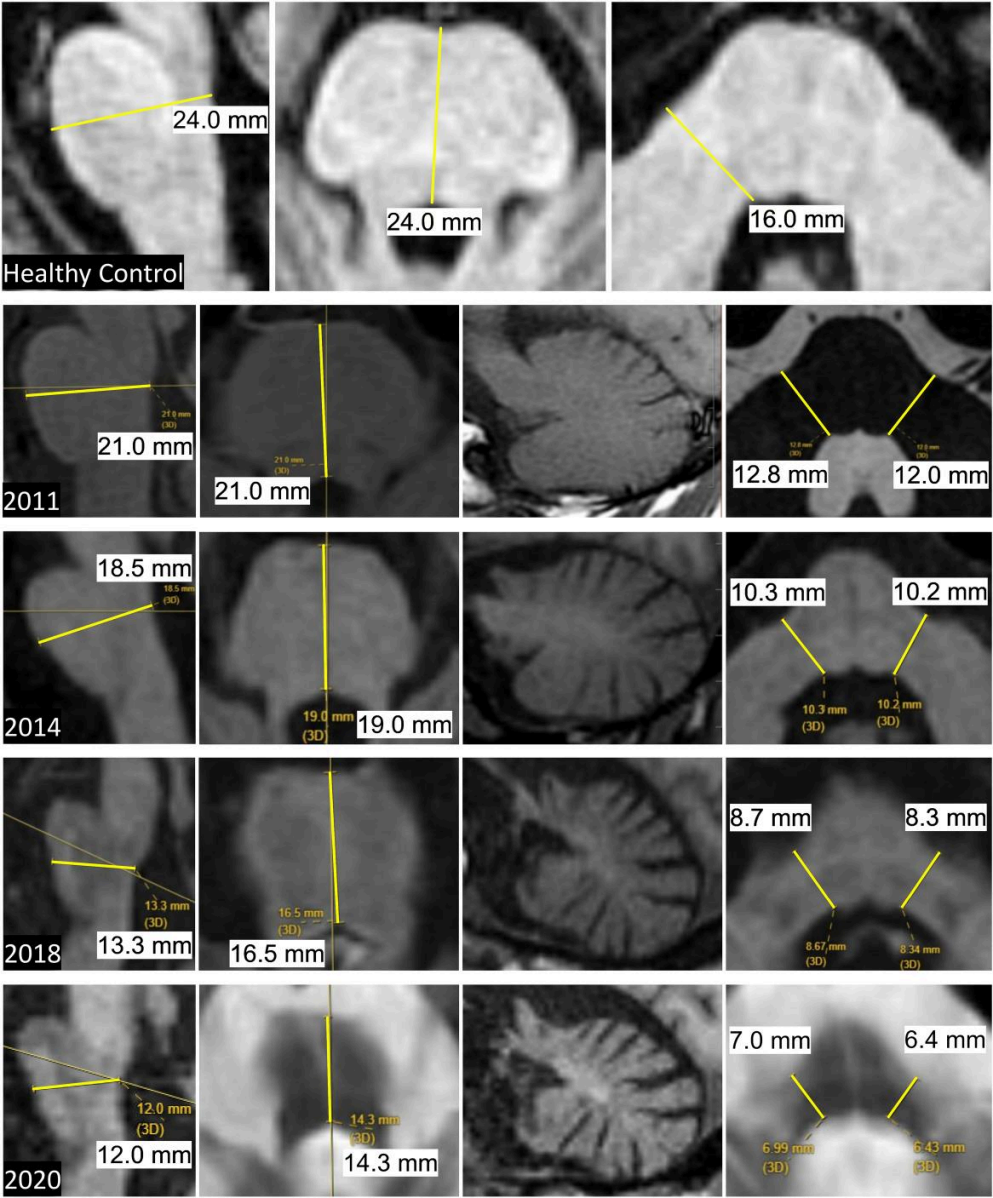
**Pons and MCP measures in MSA-C.** *Top* row: sagittal measurements (left image) and axial measurements (*middle* and right images) of the AP Pons diameter and transverse MCP diameters in a healthy control. *Bottom* four rows: sequential images from 2011 to 2020 in a patient with MSA-C showing progressive pons and cerebellar atrophy. Note that the AP Pons diameter was ascertained from the axial plane of section only in 2011. In subsequent scans, the axial plane was not perpendicular to the long axis of the brainstem and so the more accurate measure was derived from the sagittal images that was at or close to the midline as seen in the corresponding axial sections. The parasagittal views of the cerebellar hemisphere show the progressive cerebellar atrophy with shrinkage of the entire hemisphere, loss of the corpus medullare and prominence of the cerebellar folia. AP, anteroposterior; MCP, middle cerebellar peduncle; MSA-C, multiple system atrophy of the cerebellar type. Measurements in small text from screen shots of the patient’s MRI are shown in black font on white background for readability.

Measurements were derived as described below, and as shown in Fig. 1 that includes representative images of sequential MRI scans performed in a single individual with MSA-C over a period of 9 years.

#### Anteroposterior (AP) Pons diameter

We determined that it is possible to measure the pontine AP diameter accurately by paying careful attention to the imaging parameters in each case. The axial and sagittal images are viewed side-by-side in the imaging program, with the cross-referencing tool a valuable guide.

#### AP Pons diameter measured using the axial view

The mid-pons in the rostro-caudal dimension is identified in the axial plane with reference to the corresponding sagittal image. The midline AP diameter of the pons is then measured on this image as follows. A line is drawn with the ruler tool, starting at the posterior/dorsal boundary of the pons demarcated by the reliable indentation of the 4th ventricle, and extending forwards to the anterior/ventral boundary of the pons, targeting the midline where there is a reliable concavity between the two sides of the pons.

#### AP Pons diameter measured using the sagittal view

The pontine midline is identified in the sagittal plane using the corresponding axial view as a guide. A line is then drawn on the mid-sagittal section anchored at a point on the posterior boundary of the pons that forms the apex of an obtuse angle in the curvature of the pons as one descends from superior to inferior. The AP diameter of the pons is measured from this posterior location to a point on the anterior boundary of the pons, ensuring that the line is perpendicular to the long axis of the brainstem.

#### Transverse diameter of the MCPs

The axial view is used to identify the MCPs. The optimal section is between the 5th and 7th/8th cranial nerves, where the MCP merges with the pons forming an obtuse angle of ∼100° between the posterior boundary of the pons and the medial border of the MCP. The line is drawn obliquely forward from the apex of this angle, perpendicular to the long axis of the MCP.

#### Practical considerations

We recognized and addressed technical considerations in the measurements performed on these clinically derived scans. First, the mid-sagittal plane is not always exactly mid-sagittal. The ventral aspect of the pons is indented in the midline, so measurement of the AP Pons diameter on an off-centre sagittal image produces a spuriously large result. Second, the axial slice is not always aligned perpendicular to the long axis of the brainstem, so measurement on such an axial image also produces a spuriously large AP Pons diameter. While axial sequences are preferred, if we encounter either of these measurement issues, we use the AP diameter in the sagittal or axial plane that most closely approximates what we determine to be the true AP diameter, i.e. the AP diameter at the mid-rostro-caudal level of the pons, perpendicular to the long axis of the brainstem, exactly at the midline. When measuring the MCPs, we prefer high-resolution scans at 2 mm slice intervals. On 5 mm slices, there are usually two levels in which MCPs are well defined, and we use the more rostral level where the MCP transverse diameter is wider. We maintain consistency in how we measure the brainstem in the same patient over time.

### Statistical analyses

Statistical analysis was conducted using R 4.1.0 (R Foundation for Statistical Computing, Vienna, Austria) for the Exploratory cohort and SAS 9.4 (Cary, NC) for the Validation cohort. Tests for all statistical hypotheses were performed at the 0.05 two-tailed significance level. For tests on means and correlations, we used *t*-tests and Pearson correlation tests, respectively, throughout.

#### Intra-rater and inter-rater reliability

We investigated the accuracy and reproducibility of these measurements by testing intra-rater and inter-rater reliability among the senior author and three additional trained raters. Fifteen scans (five each for MSA-C, non-MSA cerebellar disease and controls) were evaluated twice by the four evaluators who were blinded to the results of the other raters. Variability for sagittal and axial pons and right and left MCP measurements were decomposed into between-subject, between-evaluator (within-subject) and residual variants using mixed-model regression analysis. The results were summarized using intraclass correlation coefficients (ICC) and comparing percent variability attributable to the subject and evaluator.

#### Correlation of pontine AP diameter with pontine volume

We used the PACS imaging system and Vitrea Web program to derive pontine volumes in 22 ataxia patients (18 MSA-C, two ILOCA, one SCA6, one FA). We outlined the pons in a series of axial and sagittal slices within the same plane, calculated the volume and correlated this with the corresponding sagittal and axial AP Pons measures.

#### Rates of change over time

We analysed pons and MCP diameters (right and left MCP measures averaged) at baseline and determined their rate of change over time, comparing them across Possible, Probable and Definite (autopsy-conformed) MSA-C and all other ataxias. In the Exploratory cohort, we included all 88 patients, 60 (68.2%) of whom had between two and six total scans over the course of their disease. In the Validation cohort, 44 of 49 patients (89.8%) had repeat scans, and we determined the rate of change by comparing the first to the last scans. We also assessed for differences within the synucleinopathies by comparing measurements in Possible/Probable MSA-C versus Probable MSA-P, and MSA-P versus PD. We used mixed-model regression for analyses with repeated measures, AP Pons and MCP diameter as a function of years since symptom onset, with subject as a random intercept, adjusting for age and sex. The SD for the rates of atrophy for MSA for both AP Pons and MCP was estimated using random-slope mixed-model regression. We considered both linear and higher-order polynomial models for years since onset.

Receiver operating characteristic (ROC) curves, and areas under these curves (AUC), were used to illustrate the performance as diagnostic markers of the pons and MCP measures as functions of threshold.

#### Prognosis in MSA-C: survival time, AP Pons diameter at time of death, residual life

We determined the length of survival in the 86 MSA-C patients who died during the course of this two decade study in both the Exploratory (*n* = 73) and Validation cohorts (*n* = 13), with disease onset measured from the first motor symptom (gait ataxia, imbalance, dysarthria, dysgraphia). We also explored predicted AP Pons diameter at time of death, and we used a linear regression model to test whether it is possible to predict residual life/survival from duration of disease and axial pons measurements.

#### Other measures

We calculated the sagittal midbrain:pons ratio according to Massey *et al*.,^33^ a useful parameter in PSP, to test our hypothesis that this ratio increases as MSA-C progresses; and we determined the axial MCP:pons ratio to test our hypothesis that this ratio remains constant as MSA-C progresses.

## Results

### Normative data for the AP Pons and transverse MCP diameters

The mean axial AP Pons diameter (mean ± SD) was 23.6 ± 1.6 mm, the sagittal AP Pons diameter was 23.8 ± 1.4 mm, and the mean MCP diameter was 16.4 ± 1.4 mm. The results are shown by decade in Table 3, together with the number of SDs below the mean for each. These measures are essentially constant through the life span within a range of ∼1 mm. The single healthy control in the 9th decade is ∼1 SD below the mean for the cohort of 72 individuals ages 10 to 79.

### Intra-rater and inter-rater reliability

For the axial AP Pons measures, the intra-rater reliability (ICC) was 0.92, and the inter-rater reliability was 0.89; and for the sagittal AP Pons measure, the intra-rater reliability was 0.86, and the inter-rater reliability was 0.76. For the MCP measure, the intra-rater reliability was 0.83 (L-MCP) and 0.78 (R-MCP), and the inter-rater reliability was 0.78 (L-MCP) and 0.71 (R-MCP).

### Diameter versus pontine volume correlations

There was strong correlation between the AP Pons diameter and the volume of the pons in both the sagittal (*r* = 0.94) and axial planes (*r* = 0.94), both *P* < 0.0001. There was a similarly strong correlation between the mean MCP measures and pons volume (*r* = 0.96, *P* < 0.0001). The volume of the pons can be predicted from the pons axial measurement with the following equation: Pons_Volume(axial)_ = [0.96 ∗ Pons_Axial_ − 8.51] mm^3^ (*R*^2^ = 88%). The corresponding equation using sagittal measures was Pons_Volume(sagittal)_ = [1.04 ∗ Pons_Sagittal_ − 10.07] mm^3^ (*R*^2^ = 88%).

### AP Pons and MCP diameters at first scan

#### Exploratory cohort

Demographic details and AP Pons and MCP parameters at initial scan are shown in Table 1.

AP Pons diameters and MCP diameters were statistically indistinguishable between the Possible/Probable and Definite MSA-C groups.

At first scan, mean axial AP Pons diameter in Possible/Probable/Definite MSA-C (*n* = 88, 19.3 ± 2.6 mm) was significantly smaller than healthy controls (*n* = 73, 23.6 ± 1.6 mm, *P* < 0.0001), ILOCA (*n* = 15, 22.1 ± 2.3 mm, *P* = 0.0002) and all other cerebellar ataxias (*n* = 78, 20.7 ± 2.6 mm, *P* = 0.0006).

Mean MCP diameter at initial scan in Possible/Probable/Definite MSA-C was 12.0 ± 2.6 mm (*n* = 82 [MCP transverse diameters could not be measured with certainty in six cases related to the plane of section]), significantly smaller than healthy controls (*n* = 73, 16.4 ± 1.4 mm), ILOCA (*n* = 15, 15.1 ± 2.2 mm) and all other cerebellar ataxias (*n* = 77 [MCP transverse diameter could not be measured with certainty in one case], 14.3 ± 2.1 mm), all *P* < 0.0001.

#### Validation cohort

Demographic details, axial AP Pons and MCP parameters for the different diagnoses at initial scan are shown in Table 2. There was no difference between Possible and Probable MSA-C in AP Pons diameter (*P* = 0.42) and mean MCP diameter (*P* = 0.29). Mean ± SD AP Pons diameter in Possible/Probable MSA-C (*n* = 49) 19.9 ± 2.1 mm was smaller than other cerebellar ataxias (*n* = 99) 21.1 ± 2.1 mm (*P* = 0.0007) and Probable MSA-P (*n* = 13) 21.5 ± 1.2 mm (*P* = 0.0008). Similarly, mean MCP diameter in Possible/Probable MSA-C (*n* = 49) 13.6 ± 2.1 mm was smaller than other ataxias (*n* = 99) 15.1 ± 1.8 mm (*P* < 0.0001) and Probable MSA-P (*n* = 13) 15.9 ± 1.4 mm (*P* = 0.0004).

Mean axial AP Pons diameter was smaller in MSA-P (*n* = 13) than in PD (*n* = 79), 21.5 ± 1.2 versus 22.9 ± 1.4 mm, *P* = 0.001. Similarly, mean MCP diameters were smaller in MSA-P (*n* = 13) than in PD (*n* = 79), 15.9 ± 1.4 versus 17.5 ± 1.1 mm, *P* < 0.0001.

In the large, unselected movement disorders subset of the Validation cohort excluding MSA-C, MSA-P, other ataxias and atypical parkinsonism (*n* = 314), at first visit, there was no clear direction of difference in AP Pons and MCP measurements. The AP Pons was smaller in patients compared to controls (22.6 ± 1.5 versus 23.6 ± 1.6 mm, *P* < 0.0001), whereas MCP transverse diameters were larger (17.1 ± 1.2 versus 16.4 ± 1.4 mm) *P* = 0.0001. The same held true for a subset including only FND/drug-induced/non-neurological cases (*n* = 108): at first visit, the AP Pons was smaller in patients than controls (22.6 ± 1.6 versus 23.6 ± 1.6 mm, *P* < 0.0001), and MCP transverse diameters were larger (17.0 ± 1.3 mm versus 16.4 ± 1.4 mm) *P* = 0.006 (Supplementary Tables 1 and 2).

### Rate of change over time of AP Pons diameters and MCP diameters

#### Exploratory cohort

**Figure 2:**
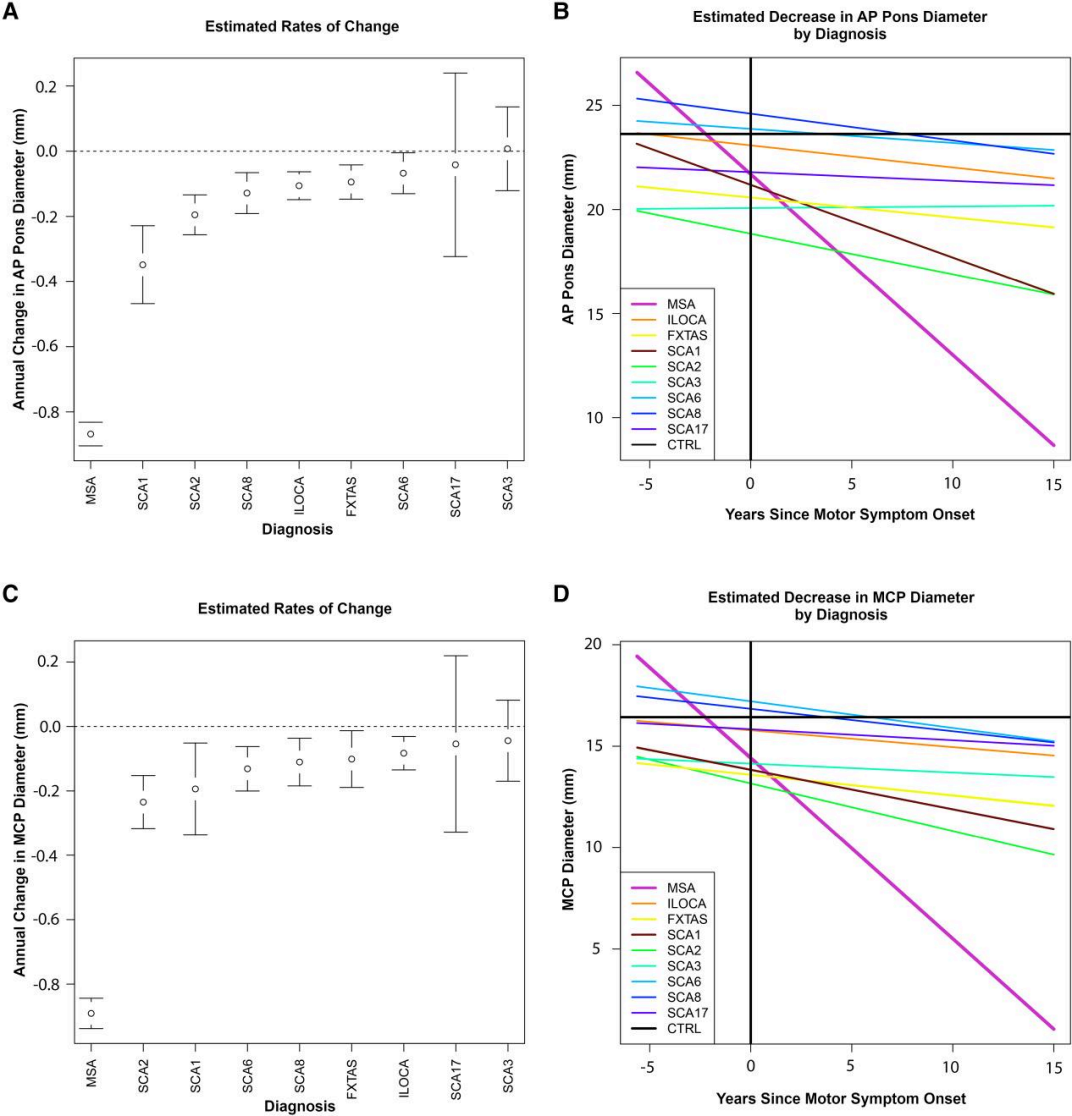
**Rates of decline of pons and MCP diameters in MSA-C versus other ataxias.** Estimated change over time in the Exploratory cohort for AP Pons and MCP diameters, derived from a mixed-model regression with subject as random effect. (**A**) Whisker plot for AP Pons. (**C**) Whisker plot for MCPs, averaged across both MCPs for each diagnosis. (**B**) Line graphs for AP Pons. (**D**) Line graphs for MCPs. AP, anteroposterior; Ctrl, healthy controls; MCP, middle cerebellar peduncle; MSA-C, multiple system atrophy of the cerebellar type; ILOCA, idiopathic late-onset cerebellar ataxia; SCA, spinocerebellar ataxia; FXTAS, fragile X-associated tremor/ataxia syndrome. The number of patients in each diagnostic category is listed in Table 1.

Whisker plots for rate of change in AP Pons and MCPs in MSA-C versus other ataxias and line graphs showing predicted initial dimensions and change over time are shown in Fig. 2.

There was no difference in annual rate of change in AP Pons and MCP diameters between Possible/Probable (*n* = 74) and Definite (*n* = 14) MSA-C cases.

Using a mixed-model regression analysis, with subject as a random effect, and fixed effects including time since onset and diagnosis, the rate of decline (mean ± standard error,SE) in axial AP Pons diameter in Possible/Probable/Definite MSA-C (*n* = 88) was −0.87 ± 0.04 mm/year, different from all other ataxias as a group (*n* = 78, −0.09 ± 0.02 mm/year), *P* < 0.0001, and individually.

The mean ± SE MCP change per year for Possible/Probable/Definite MSA-C (*n* = 88) was −0.84 ± 0.05 mm/year, also different from all other ataxias as a group (*n* = 78) −0.08 ± 0.02 mm/year, *P* < 0.0001, and individually.

The SD for the change of diameter in the MSA-C cohort was 0.28 mm/year for AP Pons measure, 0.35 mm/year for the MCP. It was not possible to compute SD for the non-MSA diagnoses because of the small numbers of individuals with repeat scans.

**Figure 3:**
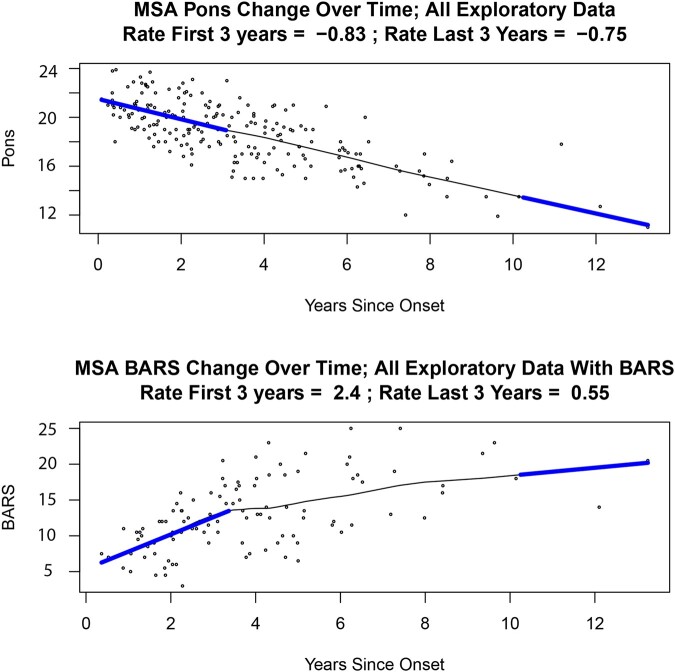
**Scatter plots showing change over time of pons diameter and BARS scores in MSA-C.** Scatter plots of rate of change over time of (**A**) AP Pons diameter and (**B**) BARS scores in the Exploratory cohort. There were 88 patients with MSA-C in the Exploratory cohort. Of these, 31 were in the early stage of the disease (0–3 years), and two patients were in the late stage of the disease (10–13 years). AP, anteroposterior; BARS, Brief Ataxia Rating Scale; MSA-C, multiple system atrophy of the cerebellar type.

We compared the rate of change in AP Pons and MCP diameter in early versus late stages of MSA-C. There was no difference in the rate of change of the AP Pons diameter during the early phase of the disease, years 0–3 (−0.81 mm/year, *n* = 31 patients) and the late stage of disease, years 10–13 (−0.75 mm/year, *n* = 2 patients) (Fig. 3A).

Given the critical clinical need specifically to differentiate MSA-C from ILOCA, both of which are sporadic ataxias without a family history, we re-fit the mixed-model regression used in the above rate comparison, using Exploratory cohort cases with diagnoses of MSA-C (Possible/Probable, Definite, *N* = 88) and ILOCA (*N* = 15). The estimated average annual rate of pons atrophy from this analysis was −0.11 mm/year for ILOCA versus −0.86 mm/year for MSA, a difference that is highly statistically significant (T = −13.4 with 200 df, Cohen’s-*d* effect size of 0.95, *P* < 0.0001), and sufficiently large to be of obvious clinical importance. For the MCP, the estimated mean annual rates of decline are −0.08 and −0.89, for ILOCA and MSA, respectively, (T = −11.3, 211 df, Cohen’s-*d* effect size of 0.78).

#### Validation cohort

Possible (*n* = 18) and Probable MSA-C (*n* = 26) were indistinguishable in their mm/year rate of change of pons and MCP diameters: AP Pons diameters −0.87 ± 0.34 versus −0.90 ± 0.57, *P* = 0.86; MCP diameters −1.03 ± 0.47 versus −0.88 ± 0.74, *P* = 0.45; see Supplementary Table 3 and Supplementary Fig. 1. In Possible/Probable MSA-C (*n* = 44), mean ± SD AP Pons diameter decreased by −0.89 ± 0.48 mm/year, faster than in other ataxias (*n* = 49) −0.10 ± 0.21 mm/year, *P* < 0.0001, and MSA-P (*n* = 9) −0.26 ± 0.42 mm/year, *P* = 0.0007. Similarly, mean MCP diameter in Possible/Probable MSA-C (*n* = 44) declined by −0.94 ± 0.64 mm/year, faster than other ataxias (*n* = 49), −0.11 ± 0.27 mm/year, *P* < 0.0001, and MSA-P (*n* = 9) −0.40 ± 0.40 mm/year, *P* = 0.02.

There was a trend towards a difference in the rate of change of axial AP Pons diameter between MSA-P (*n* = 9, −0.26 ± 0.42 mm/year) and PD (*n* = 24, −0.004 ± 0.29 mm/year), *P* = 0.06 and similarly, in MCP diameter between MSA-P (*n* = 9, −0.40 ± 0.40 mm/year) and PD (*n* = 24, −0.10 ± 0.4 mm/year), *P* = 0.07.

We evaluated Cohen’s-*d* for a mean difference in rates of atrophy between MSA-P (*n* = 13) and MSA-C (*n* = 49). The effect sizes are of the form *t*-statistic/square root(df + 1), where df + 1 is the effective sample size for the regression coefficient. The effect size for the pons was 0.35 and for the MCP (averaging left and right), 0.16. Cohen considered an effect size of 0.5 to be moderate, so these are all small effects, although the effect size is substantially larger for the pons than the MCP and is near the threshold for a moderate effect size. Note that these are average differences between the groups and do not address the question of whether this measure would be useful as a diagnostic tool in an individual patient.

#### Evolution of BARS scores over time

The range of BARS scores throughout the disease course in patients with Possible/Probable/Definite MSA-C in the Exploratory cohort was 3–25. This range reflected very early disease in some in whom the diagnosis was made with clinical certainty as the course evolved, and near-maximal severity in others. The BARS rate of change (mean ± SD) was 1.4 ± 0.17 points/year. The slope of change was non-linear, however, flattening out as the disease became more severe (Fig. 3B). The estimated rate of BARS worsening at the beginning of the course (in years 0–3, *n* = 31 individuals) was 2.4 BARS points/year, but much slower in the two individuals evaluated in years 10–13, worsening by 0.56 BARS points/year. This apparent slowing of progression late in the course reflects the ceiling effect of the BARS once patients are severely affected. This relationship holds true for other rating scales that are complicated by floor and ceiling effects at both ends of the scales.^34^

#### BARS score as a reflection of AP Pons measure

Performance on the BARS correlated with the axial AP Pons diameter at the time of the first scan (*r* = −0.66, *P* < 0.0001). The correlation of BARS score with AP Pons measures over time was somewhat less robust (*r* = −0.57 [95% CI −0.33, −0.74]), consistent with the slower rate of BARS score change late in the disease.

### Sensitivity/specificity analyses for cut-off rates of AP Pons and MCP decline

**Figure 4:**
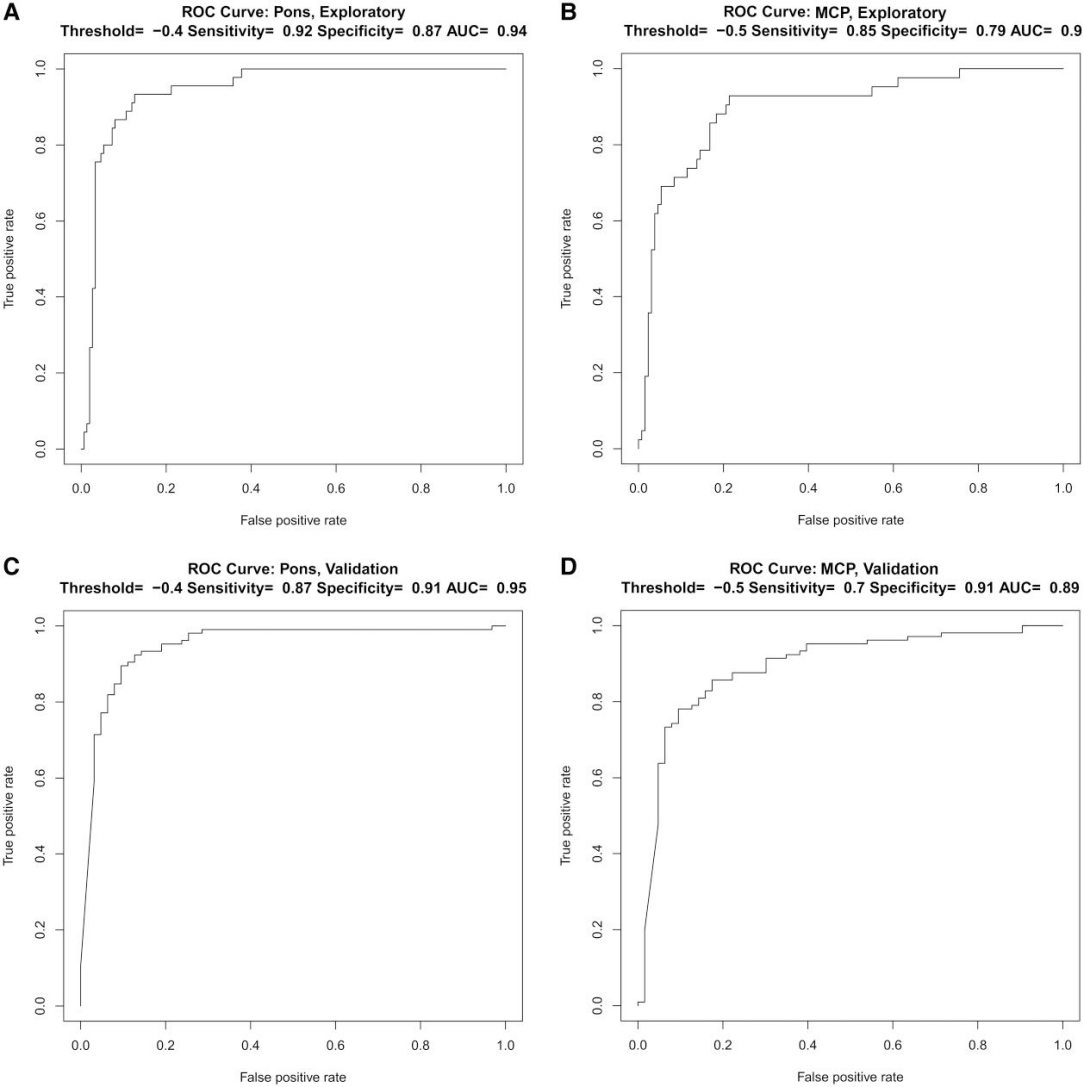
**ROC curves for predicting the diagnosis of MSA-C.** ROC curves for the prediction of MSA-C in (**A**, **B**) the Exploratory cohort and in (**C**, **D**) the Validation cohort using a threshold of −0.4 mm/year for the AP Pons and −0.5 mm/year for the MCPs. AP, anteroposterior; MCP, middle cerebellar peduncle; MSA-C, multiple system atrophy of the cerebellar type; ROC, receiver operating characteristic; AUC, area under the curve.

With an average annual rate of pons diameter decrease of −0.87 mm in MSA-C, approximately half of the MSA-C patients had slower rates of atrophy than this, but their rate of change was still much faster than that in ataxia patients who did not have MSA-C. A cut-off of −0.87 mm is therefore not appropriate for separating MSA-C from other ataxias because it would have very high specificity, but much lower sensitivity in the range of ∼50%. We found that by using both the Exploratory and the Validation cohorts to analyse sensitivity/specificity and ROC curves using specific cut-offs for AP Pons and MCP decline in MSA-C compared to all other ataxias (Fig. 4), we were able to considerably increase sensitivity to detection of MSA-C with only modest decrease in specificity. Thus, in the Exploratory cohort, using a threshold of AP Pons decline of −0.4 mm/year, we achieved sensitivity 0.92 and specificity 0.87 (AUC =0.94). Similarly, for the MCP, a threshold decline of −0.5 mm/year yielded sensitivity 0.85 and specificity 0.79 (AUC = 0.90). These results were replicated in the Validation cohort, using the same pons decline cut-off of −0.4 mm/year (sensitivity = 0.87, specificity = 0.91, AUC = 0.95) and MCP cut-off −0.5 mm/year (sensitivity = 0.70, specificity = 0.91, AUC = 0.89). This demonstrates that even when using pons/MCP diameter rates of decline that are far from our mean values, the sensitivity/specificity for predicting MSA-C versus other ataxias remains extremely high.

#### Prognosis in MSA-C: survival time, AP Pons diameter at time of death, residual life

The mean length of survival in the 86 patients who died in both the Exploratory and Validation cohorts, from first motor symptom onset to death, was 8.3 ± 3.1 years (range 3.2–16.1 years). For the 86 patients who died in both the Exploratory and Validation cohorts, the time between last MRI and death (mean ± SD) was 3.7 ± 2.5 years (Exploratory) and 2.7 ± 1.6 years (Validation). AP Pons measures at the time of the most recent scan were 17.4 ± 2.4 mm (Exploratory) and 17.3 ± 1.9 mm (Validation). These differences between the cohorts were not significant (time interval MRI to death, *P* = 0.08; AP Pons measure on last MRI, *P* = 0.87).

With a mean linear rate of decline of AP Pons diameter of −0.87 mm/year, the mean predicted AP Pons diameter at time of death was 13.6 ± 3.2 mm (25th percentile measure 11.5 mm, 75th percentile 15.8 mm).

The large SD (3.2 mm) in the predicted mean AP Pons diameter at time of death made it impractical to use this value to predict residual life for an individual patient. This was confirmed by the finding that in the 86 patients who died, there was poor correlation between actual residual life, and predicted residual life calculated using the AP Pons measure, 0.18, *P* = 0.1.

### Pons, MCP and midbrain ratios

#### MCP:pons ratio

At first scan, the mean ± SD MCP:pons ratio was minimally smaller in MSA-C compared to other diseases in both the Exploratory and Validation cohorts; Exploratory cohort: MSA-C (*n* = 82, 0.62 ± 0.08); controls (*n* = 73, 0.70 ± 0.06, *P* < 0.0001), all other cerebellar ataxias (*n* = 77, 0.69 ± 0.06, *P* < 0.0001), ILOCA (*n* = 15, 0.68 ± 0.05, *P* = 0.004); Validation cohort: Possible/Probable MSA-C (*n* = 49) 0.68 ± 0.06, all other cerebellar ataxias (*n* = 99) 0.71 ± 0.05, *P* = 0.0006. In both cohorts, there was no difference between Possible, Probable and Definite MSA-C (*P* = 0.3). In the Validation cohort, the MCP:pons ratio in MSA-C was smaller than in MSA-P (*n* = 13) 0.74 ± 0.06, *P* = 0.003.

The annual rate of change of the MCP:pons ratio could be calculated in a subset of patients. Whereas all 88 patients in the Exploratory cohort were included in the mixed-model regression analysis for rate of change of AP Pons and MCP measures, for the MCP:pons ratio, there were 53 of 60 patients with repeat scans suitable for analysis. Of the 78 patients with other ataxias, 19 had first and last scans suitable for this analysis. These images revealed a minimal annual change in the MCP:pons ratio over time: MSA-C (*n* = 53, −0.037 ± 0.030/year), other ataxias (*n* = 19, −0.004 ± 0.012/year), *P* < 0.0001. This minimal change was also seen in the Validation cohort: MSA-C (*n* = 44, −0.018 ± 0.030/year), other ataxias (*n* = 49, −0.0021 ± 0.012/year), *P* < 0.002.

#### Midbrain:pons ratio

In the Validation cohort, the mean ± SD midbrain:pons ratio at first scan was higher in Possible/Probable MSA-C (*n* = 47; 2 scans did not have corresponding sagittal sequences), at 0.80 ± 0.11 (range 0.66–1.0) than in other ataxias (*n* = 99, 0.72 ± 0.09, *P* < 0.0001) and MSA-P (*n* = 13, 0.67 ± 0.05, *P* < 0.0001). This difference was exaggerated in late-stage MSA-C when measured on the last available scan, at which time the ratio had increased to 0.90 ± 0.14 (range 0.6–1.19), reflecting preservation of the midbrain in the face of progressive atrophy of the pons.

### Sex differences in pontine AP diameter in non-ataxia neurological patients

In the non-MSA/ataxia/atypical parkinsonism cohort (*n* = 314), there was a ∼0.8 mm sex difference in AP Pons diameter (males 23.0 ± 1.5 versus females 22.2 ± 1.5 mm) and MCP diameter (males 17.5 ± 1.1 versus females 16.7 ± 1.2 mm), both *P* < 0.0001 (Supplementary Table 1). This difference remained in the more limited FND/drug-induced/non-neurological cohort (*n* = 108): AP Pons (males 23.3 ± 1.5 versus females 22.1 ± 1.4 mm), MCP (males 17.7 ± 1.2 versus females 16.6 ± 1.3 mm), both *P* < 0.0001 (Supplementary Table 2). This sex difference was not present in MSA-C, MSA-P, and atypical parkinsonism. Similarly, in the Exploratory MSA-C cohort, there was no sex difference in AP Pons or MCP transverse diameters.

## Discussion

The hypothesis of this study was that clinical brain MRI can advance the diagnosis of MSA-C during life, distinguishing MSA-C with certainty from all other causes of cerebellar ataxia. We demonstrate for the first time the critical observation that rate of decrease in the diameter of the pons and MCPs in MSA-C is uniquely rapid, faster than in any other ataxia or neurological disease in our cohorts. We therefore introduce this measure as a simple and powerful imaging biomarker for the diagnosis and progression of MSA-C.

We tested our hypothesis by measuring progressive loss of diameter of the AP Pons and MCPs in MSA-C as our primary outcome measures in large Exploratory and Validation cohorts (total MSA-C, *n* = 137 patients) in a single centre over 20 years and compared the findings with those in other cerebellar and non-cerebellar neurological disorders. We also conducted an exploratory analysis of these measures in a small number of MSA-P patients compared to PD.

We determined normative values for the AP diameter of the pons and the transverse diameter of the MCPs in a healthy cohort, consistent with previous findings.^16^ We confirmed the validity of diameter measurements by showing tight correlations with volumetric assessments of the pons, and demonstrated intra-rater and inter-rater reliability in the measurements of these cardinal parameters.

In the Exploratory and Validation cohorts, we show that at first MRI in a patient with MSA-C, the pons and MCP diameters are significantly smaller than in all the other ataxias we investigated, collectively and individually, including SCA types 1, 2, 3, 5, 6, 7, 8 and 17, ILOCA, FA and FXTAS. In some SCAs, such as SCA2, marked olivopontocerebellar atrophy develops as the disease progresses, so comparison of imaging findings at any single time-point in an individual patient must be considered at equivalent stages of the clinical course. With this caveat, the conclusion from this study about pons and MCP dimensions in MSA-C holds true.

Further, while Possible, Probable and Definite MSA-C are defined by clinical features, we show that these stages of MSA-C are indistinguishable from each other when measuring rate of change over time of the AP Pons and MCP transverse diameters. The AP Pons diameter declines at an average rate of 0.87 mm/year. This key radiological feature of volume loss in the pons and MCPs in MSA-C is seen in other cerebellar ataxias, previously designated olivopontocerebellar atrophies, but here we show that the rate of decline of the AP Pons and MCP diameters is uniquely faster in MSA-C. The estimated BARS rate of clinical decline in MSA-C is 1.4 mm BARS points/year when averaged over the course of the disease, and correlates with the brainstem measures, providing clinical validation of the radiological assessments. The BARS rate of worsening slows in the late stages of the disease, a consequence of the ceiling effect of the clinical scoring system in severely compromised patients.^34^ The AP Pons diameter measurement is therefore an accurate assessment tool throughout the course of disease.

In our cohorts, the duration of MSA-C from first motor symptoms to death was 8.3 ± 3.1 years, range (3.3–16.1 years). The range was more limited (4.9–14.7 years) if we excluded nine outliers. Seven patients died between 3.3 and 4.8 years, of whom four had catastrophic emotional reaction to illness and elected for early hospice, one died from septicaemia, one from traumatic brain haemorrhage and one had developed severe autonomic symptoms 10 years prior to death. There are many potential factors contributing to earlier death in MSA, such as occurs in patients with stridor who are more likely to succumb from sudden death.^35^ Two of our patients had long survival times (15.9 and 16.1 years), both of whom were elite athletes who noticed motor changes very early in their course.

In our Validation cohort, we show a trend for the pons and MCP diameters to distinguish MSA-P from PD as suggested previously.^13,36^ The small numbers in this subset of the cohort may have precluded the determination of clinical significance, and this finding will need to be further evaluated. In addition, at the group level, there were small to moderate differences in pontine more than MCP diameter changes in MSA-C versus MSA-P. Annual decline in pontine/MCP measures may prove to be useful as a metric to distinguish MSA-C from MSA-P, but given that the number of MSA-P cases in our cohort was small, the measurements were assessed at only two time-points, and some MSA-P cases were more purely parkinsonian whereas others had both parkinsonism and ataxia from the outset, this will need to be addressed in greater detail in a larger study of MSA-P.

A sex difference in AP Pons and MCP diameters was seen in the general movement disorder patients in the Validation cohort, but not in the healthy controls or other patient cohorts in this study. We did not measure total brain volume or correlate brainstem measures with cranial size and brain volume, as this was outside the scope of this study, but these ratios would be pertinent in investigations that focus on sex differences in these measures in healthy controls. The finding of a sex difference in brainstem measures needs to be replicated, but critically, for the purposes of our study and clinical question, the rate of change of the pons and MCP measures was independent of sex or the diameters of the brainstem measures at onset.

### Other imaging findings of note in MSA-C

There is a small decline in the MCP:pons ratio over time. The MCPs convey the axons of the pontine nuclei to the cerebellum, and as the pontine neurons and their axons degenerate, the volume loss in these structures occurs largely in tandem. The small decline in the ratio may reflect less atrophy of the pontine tegmentum compared to the pontine base that contains the neurons of origin of the MCP fibres. In contrast, and in agreement with Peralta *et al*.,^13^ the midbrain:pons ratios are higher in MSA-C than in other ataxias and they increase over time, a consequence of the progressive pontine atrophy compared to preservation of the midbrain.

### Comparison with the literature

Our findings are in line with recent studies that have focused on the role of imaging in the diagnosis and assessment of progression of MSA-C. Kim *et al*.^37^ compared MSA-C to SCAs and found that the HCBS and MCP hyperintensities were largely confined to MSA-C, with high individual and combined positive predictive values, but the differential value of these MRI signs decreased over time. In other studies comparing MSA-C and MSA-P, the HCBS and hyperintense putaminal rim signs were infrequently observed.^38^

The Movement Disorders Society Neuroimaging study group noted that specific features on conventional MRI suggestive of MSA-C include an increased midbrain:pons ratio, decreased Magnetic Resonance Parkinsonism Index (pontine area/midbrain area × MCP width/superior cerebellar peduncle width)^39^ typically <5, HCBS, cerebellar atrophy, MCP atrophy and the presence of T2 hyperintense signal in the MCPs, putaminal atrophy and presence of bilateral hyperintensity in the posterior half of the putamen on susceptibility weighted imaging and hyperintensity on apparent diffusion coefficient, reduced MCP diameter < 8 mm (more accurately, the MCP height).^13,40^ Dopamine transporter scans with reduced striatal uptake (not useful to differentiate from PD) and positron emission tomography with decreased metabolic activity in the basal ganglia, putamen, pons and cerebellum were also felt to be suggestive.^13,40^

Nicoletti *et al*.^41^ compared MSA to PD measured MCP diameter in the sagittal plane, and determined that mean MCP width (more accurately, height) was different from PD and healthy controls. Gama *et al*.^42^ compared MRI features in PD, MSA-C, MSA-P and PSP, assessing midbrain area, pons area and MCP and superior cerebellar peduncle (SCP) dimensions. Median MCP diameter (measured axially) was 17.1 mm in PD, 14.5 mm in PSP, 9.7 mm in MSA-C and 11.7 mm in MSA-P, consistent with our findings. SCP width was significantly reduced in PSP patients and in MSA-C, pons area below 315 mm^2^ showed good specificity (93.8%) and positive predictive value (72.7%). Kim *et al*.^37^ noted that MCP widths (assessing MCP height in the sagittal plane) were smaller and showed a greater decrease in MSA-C than in SCAs. Carré *et al*.^16^ assessed the MRI in 80 patients with ataxia (26 with MSA-C) at baseline and one-year follow-up. Hyperintensity of the MCP and the HCBS was more frequent in MSA-C and had the highest specificity (98.5%) and positive predictive value (91.7%) for MSA-C. The AP Pons diameter was different in MSA-C (20.15 ± 2.22 mm) versus other ataxias (22.00 ± 2.62 mm), as were MCP diameters in MSA-C [12.46 ± 2.77 mm (5–18 mm)] versus other ataxias (14.50 ± 1.68 mm). At one-year follow-up, pons AP diameter in MSA-C dropped to 18.27 ± 2.68 mm in MSA-C but remained static in the other ataxias, while the MCP size decreased to 10.57 ± 2.88 mm in MSA-C versus 14.53 ± 1.81 mm in other ataxias. There was no significant change in the midbrain diameter at baseline/follow-up. These findings are fully consistent with our present observations.

In the SPORTAX registry of sporadic ataxia patients with onset > 40 years,^43^ imaging findings were studied together with fluid biomarkers to distinguish patients with MSA-C from those with non-MSA sporadic adult-onset ataxia (SAOA). They found that cerebellar white matter, pons volume and a composite pons and MCP abnormality score (PMAS), together with the level of plasma neurofilament light chain, separated MSA-C from SAOA at baseline. In MSA-C, the pons-MCP score increased faster than SAOA, pons volume had the highest sensitivity to change, and the PMAS was a predictor of faster progression. These results are also fully concordant with our extended longitudinal observations.

### Limitations

In the normative dataset we used ∼10 data points for each decade for the measurements of the AP Pons and transverse MCP diameters. The concern that this may not be sufficient data to provide an accurate estimate across the lifespan is offset by the fact that the values are strikingly stable.

The method of assessing the AP Pons and MCP diameters in clinical MRI has inherent challenges. This relates to the angle of the axial plane of section, and the degree to which the mid-sagittal images are truly midline. We were aware of these difficulties in real time as the study progressed, which afforded us the opportunity to develop an approach to overcome this ubiquitous issue in clinical imaging, as described in the ‘Methods’ section. Imaging software now available can resample images in true axial and sagittal planes, but this depends on the resolution of the images, the thickness of section and the sophistication of the imaging centres. Thus, while it may be optimal to reorient or rotate MRI images to ensure head orientation standardization for manual annotation tasks such as those in this study, this facility may not always be available in the clinical setting.

We note that whereas the high resolution 3D T_1_-weighted imaging exemplified in SPGR and MPRAGE images are ideal for measurements, standard T_1_-weighted and T_2_-weighted images are entirely satisfactory for this approach. We avoid FLAIR images because of indistinctness of the boundaries of the pons and MCP in this sequence. Cognizant of these measurement challenges, we show that this approach provides reliable and reproducible results.

In the Exploratory cohort, measurements were performed to an accuracy of 0.1 mm. In the Validation cohort, measurements were performed with less granularity to an accuracy of the nearest 0.5 mm, to account for differences in measurements across different conventional imaging viewers. For example, some clinical radiology applications have a 1 mm accuracy, as in previous clinically accessible radiology viewing software at our institution. This notwithstanding, the agreement in the measurements between the two cohorts was extremely tight, attesting to the internal consistency of the approach.

Intra-rater and inter-rater reliability for determination of the AP Pons and transverse MCP measures was conducted using 15 MRI scans reviewed by four independent raters. The work survived statistical analysis and showed high intra-rater and inter-rater reliability. The numbers we used are also in line with previous studies developing imaging metrics for analysis, as in the development of the midbrain:pons ratio in PSP by Massey *et al.*,^33^ which included a single rater reviewing 21 pathological scans.

Our available data indicate that the rate of atrophy of the AP Pons and MCP are linear throughout the course of the illness. We had access to sequential imaging in only a small number of patients in late-stage disease (*n* = 2), so the strength of this conclusion must necessarily be tempered by this numerical asymmetry.

In the movement disorder cohort that did not include patients with ataxia or MSA, a sex difference was present in brainstem measures, females smaller than males. This difference was not present in the remainder of the patient cohorts, and it was not reported in the 1000 Functional Connectomes healthy control dataset. Sex differences in regional brain volumes are often explained by differences in head size, but we did not have head size measurement in the clinical studies and therefore cannot comment on this further. It is conceivable that controlling for head size may shrink the standard deviation and improve diagnostic sensitivity in the future.

Since the completion of this study, we have had the opportunity to encounter the exceedingly rare JC virus granule cell neuronopathy (JCV-GCN), which produces very rapid volume loss in the pons and MCPs similar to and perhaps even more aggressively than in MSA-C, without the typical appearance of JC virus associated progressive multifocal leukoencephalopathy.^44^ The clinical constellation of JCV-GCN is entirely different from MSA-C and the two disorders should not be confused with each other, but this occurrence emphasizes the pitfall encountered when considering a single finding to be pathognomonic of a medical condition.

### Future directions

Quantitative morphometric analyses of the pons and MCP that provide more granularity are likely to detect smaller changes over shorter periods. The measurement interval may then decrease from ≥1 year (as in this study) to a matter of months, enhancing the real-time utility of this approach for clinical care and research.

The morphometric changes in the pons are not confined to its loss of diameter and volume. The shape of the pons becomes severely distorted, with anterior pontine beaking, and asymmetry in the rostral–caudal dimension reflecting our observation that the pons does not shrink uniformly. Novel imaging techniques may shed light on the structural disintegration underlying the pontine atrophy and link the known pathology of axons and their neurons of origin with the evolution of the disease.

Our observations of diminished AP Pons diameter notable already at first visit after motor symptom onset, together with the stable rate of change of AP Pons diameter over time, have implications for even earlier diagnosis of MSA-C. RBD is a harbinger of neurodegenerative synucleinopathy,^45^ and otherwise unexplained urinary urgency is also a common early symptom. By comparison of the morphometric findings in the patient with RBD against normative databases, and documentation of an annual AP Pons rate of decline of −0.87 mm/year, or equivalent volumetric change in quantitative morphometry, we predict that it will be possible to diagnose MSA-C even before the onset of motor symptoms and the inexorable progression of the remainder of the syndrome, opening the way to future prevention of the disorder.

## Conclusions

In this 20-year longitudinal clinical and imaging study, we show that AP Pons and MCP transverse diameters are phenotypic imaging biomarkers in MSA-C. In the correct clinical context, AP Pons diameter decline of >0.4 mm/year has a sensitivity and specificity of >90% for the diagnosis of MSA-C. Further, a rate of decline of 0.87 mm/year is sufficient for the definitive diagnosis of MSA-C in an individual patient during life. This simple and powerful approach has deep implications for diagnosis, prognosis and therapy in MSA-C. It also implies the opposite conclusion, that an individual with adult-onset sporadic ataxia who does not have the prerequisite annual change of AP Pons and MCP diameter does not have MSA-C, and the search for the cause of the clinical syndrome should continue. The power of our innovation is that it enables the clinician to make the diagnosis of MSA-C with certainty, and to do this earlier than the current diagnostic criteria^15^ suggest. Our method capitalizes on a simple, ubiquitous, imaging tool, without the need for morphometric analysis and complex algorithms. It may nevertheless be useful to combine these imaging features with biomarkers like neurofilament light chain^46-48^ and novel assays of α-synuclein on skin biopsy^49^ to aid diagnosis and further characterize severity and rate of neurodegeneration. Future studies may be able to characterize and quantify these imaging observations with finer granularity and over shorter time frames.

## Supplementary Material

fcae019_Supplementary_Data

## Data Availability

All data used in this study are maintained under an IRB-approved confidential, secure, password protected MGH Ataxia Center Patient Registry. Deidentified data relevant to this manuscript that support the findings of this study are available upon reasonable request from the corresponding author. The data are not publicly available, as they include information that could compromise the privacy of research participants.
