# Supplementary material for: Rates of change of pons and middle cerebellar peduncle diameters are diagnostic of multiple system atrophy of the cerebellar type
Source: Brain Commun. 2024 Feb 21;6(1):fcae019. doi: 10.1093/braincomms/fcae019 (PMC10896291; doi:10.1093/braincomms/fcae019)
Supplement: fcae019_Supplementary_Data [file fcae019_supplementary_data.docx]

**Supplementary Material**

Supplementary Table 1.

AP Pons and MCP measures in a non-MSA/ataxia/atypical parkinsonism subset of the Validation cohort by age decade, including comparison by sex.

| All non-MSA/ataxia/  atypical parkinsonism | Total | Total | Total | Male | Male | Male | Female | Female | Female | M vs. F Pons | M vs F  MCP |
| --- | --- | --- | --- | --- | --- | --- | --- | --- | --- | --- | --- |
| Age category, y | n | AP Pons | Mean MCP | n | AP Pons | Mean MCP | n | AP Pons | Mean MCP | p | p |
| Total | 314 | 22.6±1.5 | 17.1±1.2 | 140 | 23.0±1.5 | 17.5±1.1 | 174 | 22.2±1.5 | 16.7±1.2 | **<0.0001** | **<0.0001** |
| 0-9 | 2 | 21.5±3.5 | 16.3±1.1 | 1 | 24 | 17 | 1 | 19 | 15.5 |  |  |
| 10-19 | 9 | 22.4±1.8 | 17.1±1.2 | 3 | 24.2±1.8 | 18.0±1.0 | 6 | 21.5±1.0 | 16.6±1.0 |  |  |
| 20-29 | 13 | 22.3±2.0 | 16.6±1.2 | 6 | 23.3±1.3 | 17.1±1.2 | 7 | 21.4±2.1 | 16.2±1.1 |  |  |
| 30-39 | 15 | 23.7±1.6 | 17.9±1.5 | 8 | 24.4±1.1 | 18.5±1.3 | 7 | 22.9±1.8 | 17.2±1.4 |  |  |
| 40-49 | 38 | 22.2±1.7 | 17.1±1.5 | 12 | 23.2±1.3 | 18.1±0.9 | 26 | 21.8±1.7 | 16.6±1.4 |  |  |
| 50-59 | 64 | 22.8±1.5 | 17.3±1.2 | 32 | 23.0±1.6 | 17.6±1.2 | 32 | 22.5±1.3 | 16.9±1.2 |  |  |
| 60-69 | 94 | 22.7±1.4 | 17.1±1.0 | 47 | 22.9±1.4 | 17.3±1.0 | 47 | 22.6±1.4 | 16.9±0.9 |  |  |
| 70-79 | 55 | 22.4±1.4 | 17.0±1.2 | 21 | 22.9±1.5 | 17.4±1.0 | 34 | 22.1±1.1 | 16.8±1.2 |  |  |
| 80-89 | 22 | 21.7±1.4 | 16.3±1.1 | 9 | 21.9±1.2 | 17.0±1.1 | 13 | 21.5±1.5 | 15.8±0.9 |  |  |
| 90-99 | 2 | 22.3±0.4 | 17.3±0.4 | 1 | 22 | 17 | 1 | 22.5 | 17.5 |  |  |

Abbreviations: y: years; M: Male; F: Female.

Supplementary Table 2.

AP Pons and MCP measures in a non-degenerative movement disorders subset of the Validation cohort by age decade, including comparison by sex.

| Only FND/drug-induced/non-neurological | Total | Total | Total | Male | Male | Male | Female | Female | Female | M vs. F Pons | M vs F  MCP |
| --- | --- | --- | --- | --- | --- | --- | --- | --- | --- | --- | --- |
| Age category, y | n | AP Pons | Mean MCP | n | AP Pons | Mean MCP | n | AP Pons | Mean MCP | p | p |
| Total | 108 | 22.6±1.6 | 17.0±1.3 | 38 | 23.3±1.5 | 17.7±1.2 | 70 | 22.1±1.4 | 16.6±1.3 | **0.0001** | **<0.0001** |
| 0-9 | 0 |  |  |  |  |  |  |  |  |  |  |
| 10-19 | 5 | 22.0±2.3 | 17.2±1.5 | 1 | 26 | 19 | 4 | 21.0±0.8 | 16.7±1.3 |  |  |
| 20-29 | 7 | 23.3±1.0 | 17.5±0.7 | 4 | 23.8±1.0 | 17.8±0.7 | 3 | 22.7±0.8 | 17.0±0 |  |  |
| 30-39 | 9 | 23.8±1.5 | 18.1±1.6 | 5 | 24.2±1.3 | 18.5±1.7 | 4 | 23.4±1.7 | 17.6±1.4 |  |  |
| 40-49 | 25 | 21.9±1.6 | 16.8±1.6 | 7 | 23.1±1.3 | 18.0±0.9 | 18 | 21.5±1.5 | 16.4±1.6 |  |  |
| 50-59 | 23 | 22.5±1.6 | 17.1±1.3 | 10 | 23.0±1.9 | 17.3±1.5 | 13 | 22.2±1.4 | 16.9±1.2 |  |  |
| 60-69 | 27 | 22.9±1.2 | 17.0±0.9 | 9 | 23.2±1.3 | 17.3±0.9 | 18 | 22.8±1.2 | 16.8±0.9 |  |  |
| 70-79 | 7 | 21.9±1.2 | 16.6±1.4 | 2 | 22.0±1.4 | 17.8±0.4 | 5 | 21.9±1.2 | 16.2±1.4 |  |  |
| 80-89 | 5 | 21.9±1.5 | 15.4±1.0 | 0 |  |  | 5 | 21.9±1.5 | 15.4±1.0 |  |  |

Abbreviations: y: years; M: Male; F: Female; FND: Functional neurological disorder.

Supplementary Table 3.

Validation cohort AP Pons and MCP rates of change over time in MSA-C, MSA-P, non-MSA ataxias, atypical parkinsonism and PD.

| Diagnosis | N multiple scans | Change in AP Pons/year (mean±SD), mm/year | Change in MCP/year (mean±SD), mm/year |
| --- | --- | --- | --- |
| **MSA-C Possible/Probable (n=49)** | **44** | **-0.89±0.48** | **-0.94±0.64** |
| MSA-C Probable (n=30) | 26 | -0.90±0.57 | -0.88±0.74 |
| MSA-C Possible (n=19) | 18 | -0.87±0.34 | -1.03±0.47 |
| **MSA-P (n=13)** | **9** | **-0.26±0.42** | **-0.40±0.40** |
| **All non-MSA ataxias (n=99)** | **49** | **-0.097±0.21 (-0.60 to 0.53)** | **-0.11±0.27 (-1.16 to 0.63)** |
| Genetic/likely genetic ataxia (n=72) | 34 | -0.092±0.21 | -0.14±0.25 |
| Acquired/Immune ataxia (n=12) | 8 | -0.19±0.23 | -0.13±0.32 |
| Sporadic/Ataxia NOS (n=15) | 7 | -0.0075±0.17 | -0.011±0.30 |
| Genetic ataxias (n=72, multiple scans n=34) | | | |
| SCA2 (n=5) | 3 | -0.37±0.08 | -0.29±0.21 |
| SCA3 (n=12) | 2 | -0.13±0.11 | -0.24±0.26 |
| SCA6 (n=4) | 4 | -0.24±0.14 | -0.11±0.078 |
| SCA8 (n=3) | 1 | 0 | -1.16* |
| SCA17 (n=1) | 1 | -0.37 | -0.093 |
| SCA28 (n=1) | 1 | 0.17 | 0 |
| SCA34 (n=1) | 1 | -0.22 | -0.19 |
| SCA36 (n=1) | 1 | 0 | -0.17 |
| FA/LOFA (n=9) | 5 | -0.073±0.12 | -0.023±0.11 |
| FXTAS (n=4) | 3 | 0.080±0.14 | -0.031±0.031 |
| Late-onset Tay-Sachs (n=7) | 2 | -0.029±0.041 | -0.092±0.21 |
| PNPLA6-related disorder (n=1) | 1 | 0 | -0.35 |
| CANVAS (n=1) | 1 | 0 | 0 |
| AOA4 (n=1) | 1 | 0 | 0 |
| GSS (n=1) | 1 | 0 | 0 |
| Likely genetic NOS (n=12) | 6 | -0.035±0.36 | -0.12±0.28 |
| Other disorders | | | |
| Other atypical parkinsonism (n=40) | 6 | -0.08±0.16 | -0.39±0.77 |
| **PD (n=79)** | **24** | **-0.004±0.29** | **-0.10±0.40** |

* Spurious value - technical issues as slices not the same and unusual pons/MCP configuration

Rate of change = (last available scan – first scan axial AP Pons/mean MCP dimensions, in mm) / (age at last scan – age at first scan, in years). Rate of change only calculated for individuals with multiple scans.

Abbreviations: MSA: Multiple system atrophy; MSA-C: Multiple system atrophy of the Cerebellar type; MSA-P: Multiple system atrophy of the Parkinsonian type; NOS: Not otherwise specified; SCA: Spinocerebellar ataxia; FA: Friedreich’s ataxia; LOFA: Late-onset Friedreich’s ataxia; FXTAS: Fragile X-related Tremor/Ataxia syndrome; CANVAS: Cerebellar ataxia with neuropathy and vestibular areflexia syndrome; AOA4: Ataxia with oculomotor apraxia type 4; GSS: Gerstmann-Sträussler-Scheinker disease; PD: Parkinson’s disease.

Supplementary Figure 1.

Validation cohort MSA and non-MSA ataxia rates of AP Pons and MCP change over time.

Whisker plots illustrate mean±SD rates of change (mm/year) for all diagnoses. Rate of change = (last available scan – first scan axial AP Pons/mean MCP dimensions, in mm) / (age at last scan – age at first scan, in years). Rate of change only calculated for individuals with multiple scans.

**A**

**B**

Diagnoses and sample sizes as per Supplementary Table 1, only including individuals with multiple scans:

MSA-C: Multiple system atrophy of the Cerebellar type (n=44); MSA-P: Multiple system atrophy of the Parkinsonian type (n=9); Other ataxia: All non-MSA ataxias (n=49); Genetic ataxia (n=34); SCA2: Spinocerebellar ataxia type 2 (n=3); SCA3 (n=2); SCA6 (n=4); SCA8 (n=1, MCP value excluded as spurious); SCA17 (n=1; SCA28 (n=1); SCA34 (n=1); SCA36 (n=1); FA: Friedreich’s ataxia/late-onset Friedreich’s ataxia (n=5); FXTAS: Fragile X-related Tremor/Ataxia syndrome (n=3); LOTS: Late-onset Tay-Sachs (n=2); PNPLA6-related disorder (n=1); CANVAS: Cerebellar ataxia with neuropathy and vestibular areflexia syndrome (n=1); AOA4: Ataxia with oculomotor apraxia type 4 (n=1); GSS: Gerstmann-Sträussler-Scheinker disease (n=1); Genetic NOS: Genetic ataxia, not otherwise specified (n=6); Acquired: Acquired/autoimmune ataxia (n=8); Sporadic/NOS: Sporadic ataxia or ataxia not otherwise specified (n=7).
